# *Giardia* fibrillarin: a bioinformatics exploration of sequence and structure

**DOI:** 10.1007/s13353-024-00920-w

**Published:** 2024-11-11

**Authors:** Francisco Alejandro Lagunas-Rangel

**Affiliations:** 1 Department of Genetics and Molecular Biology, Centro de Investigación y de Estudios Avanzados del Instituto Politécnico Nacional, Mexico City, Mexico; 2 Department of Surgical Sciences, Functional Pharmacology and Neuroscience, Uppsala University, Husargatan 3, BMC Box 593, 751 24 Uppsala, Sweden

**Keywords:** Nucleolus, Ribosomal RNA, Intestinal parasite, rRNA processing, Nucleolar ribonucleoprotein particle

## Abstract

Fibrillarin is a key nucleolar S-adenosyl-L-methionine (SAM)–dependent methyltransferase, highly conserved among species in both sequence and function. It is actively involved in numerous cellular processes, particularly in the early stages of pre-ribosomal RNA processing. Although a fibrillarin orthologue has been identified in *Giardia*, an intestinal protozoan parasite responsible for numerous infections worldwide, its functional and structural features in this parasite remain largely unexplored. In this study, bioinformatics tools were used to analyze the sequence and structure of *Giardia* fibrillarin to provide insights that could aid future experimental and therapeutic investigations. Taking advantage of the protein sequence of *Giardia* fibrillarin, multiple bioinformatics tools were employed to estimate its domains, nuclear and nucleolar localization signals (NLS and NoLS), post-translational modifications, phylogeny, and three-dimensional structure. The analysis revealed significant conservation of *Giardia* fibrillarin, showing close relationships with archaeal and parasitic amoeba orthologs. It retains essential features such as the SAM-dependent methyltransferase domain, a glycine- and arginine-rich domain, and both NLS and NoLS. While the core region of the protein is structurally conserved, the N-terminal region exhibits notable divergence. The potential for inhibition of *Giardia* fibrillarin by SAM competitors suggests that it could be a promising target for drug development, particularly for strains resistant to current treatments.

**Supplementary Information:**

The online version contains supplementary material available at 10.1007/s13353-024-00920-w.

## Introduction

*Giardia duodenalis*, an anaerobic flagellated binucleate protozoan, is the causative agent of more than 300 million cases of diarrheal disease worldwide, with a particularly high impact in developing and low-income countries (Cernikova et al. 2018). This organism belongs to the order Diplomonadida in the Metamonada group within the supergroup Excavata (Burki et al. 2020). Notably, *Giardia* is characterized by an exceptionally compact genome (12.6 Mb) (Xu et al. 2020), marked by a significant reduction in the components of various cellular processes, including the absence of some organelles typical of other eukaryotic cells, such as the classical Golgi apparatus, peroxisomes, and mitochondria (Cernikova et al. 2018). *Giardia* has a simple life cycle consisting of two main phases: the trophozoite, a vegetative and motile form, and the cyst, an environmentally resistant and highly infective form (Einarsson et al. 2016).

On the other hand, in eukaryotic cells, the nucleolus is the most prominent nuclear body, constituting a functional and biophysically distinct compartment, mainly responsible for the assembly of ribonucleoprotein particles (RNP) crucial in ribosome biogenesis (Boisvert et al. 2007). In most eukaryotes, the nucleolus is organized into three distinct compartments known as the fibrillar center (FC), dense fibrillar component (DFC), and granular component (GC). Each compartment has specific responsibilities related to ribosomal biogenesis. For example, the FC participates in rRNA transcription, the CFD manages the processing of ribosomal subunits, and the GC serves as the site of ribosomal subunit assembly (Tiku and Antebi 2018). Numerous proteins have been identified in the nucleolus, but one that stands out for both its concentration and important functions is fibrillarin (El Hassouni et al. 2019). Fibrillarin is an essential nucleolar protein whose sequence and function have been conserved throughout evolution (Rodriguez‐Corona et al. 2015). As an S-adenosyl-L-methionine (SAM)–dependent methyltransferase, fibrillarin plays a crucial role in both RNA and protein methylation. Furthermore, it is a key component of small nucleolar ribonucleoproteins (snoRNPs) and contributes significantly to pre-ribosomal RNA (rRNA) methylation, processing, and pre-ribosome assembly (Shubina et al. 2016).

Notably, *Giardia* show a singular small granular nucleolus, approximately 20 to 50 nm, located at the periphery of each nucleus (Tian et al. 2010). Despite its potential significance, the *Giardia* nucleolus has received relatively little attention and remains poorly understood (Lagunas-Rangel 2023). Similarly, while an orthologue of fibrillarin has been identified in *Giardia* (Narcisi et al. 1998a), research into this protein has been limited. As a result, the specific structural and functional characteristics of fibrillarin in this parasite remain largely unexplored. In this sense, the main objective of this study was to perform a comprehensive analysis of the sequence and structure of *Giardia* fibrillarin using bioinformatics methods. The information derived from this analysis has identified several key characteristics and potential functions of this protein, which could help to guide future research.

## Materials and methods

### Sequence analysis

The *Giardia* fibrillarin sequence (GL50803_97219) was obtained from the *Giardia* database (GiardiaDB) (McArthur 2000). The physicochemical parameters of *Giardia* fibrillarin were examined by ExPASy ProtParam (Gasteiger et al. 2005). Identity and similarity comparisons were performed with CLC Genomics Workbench 23 (QIAGEN Digital Insights). Structural domain analysis was performed using InterPro (Blum et al. 2021) and PROSITE (Hulo et al. 2006), applying predefined parameters. For nuclear localization signal (NLS) prediction, NLStradamus (Nguyen Ba et al. 2009) was used with default parameters. To predict the nucleolar localization signal (NoLS), it was used the NoD server (Scott et al. 2011). A threshold score was set to 0.6, instead of the default 0.8, based on Giardia fibrillarin sequence divergence and prediction data. Phosphorylation site prediction was performed using GPS 6.0 (Chen et al. 2023), NetPhos 3.1 (Blom et al. 1999), and PhosphoSVM (Dou et al. 2014). In addition, acetylation site prediction was conducted using GPS-PAIL 2.0 (Deng et al. 2016), while methylation site prediction was carried out with PRmePRed (Kumar et al. 2017) with default parameters. Protein–protein interaction networks functional enrichment analysis was performed using the STRING database with predefined parameters (Szklarczyk et al. 2021).

### Phylogenetic analysis

Sequences of fibrillarin orthologs in 58 different species, including mammals, birds, fish, amphibians, reptiles, insects, worms, fungi/yeasts, protozoa, and archaea, were obtained from the UniProt knowledgebase (UniProtKB) (Bateman et al. 2021). In addition to the *Giardia* fibrillarin sequence, the following orthologs were also considered: *Halobacterium salinarum* (Q9HQG3), *Archaeoglobus fulgidus* (O28192), *Thermococcus kodakarensis* (Q5JFN1), *Pyrobaculum aerophilum* (Q8ZTI9), *Saccharolobus solfataricus* (P58032), *Spironucleus salmonicida* (SS50377_27681), *Leishmania mexicana* (LmxM.36.3070), *Leishmania major* (P35549), *Trypanosoma vivax* (TvY486_1007310), *Trypanosoma rangeli* (TRSC58_02920), *Entamoeba histolytica* (EHI_118840), *Entamoeba dispar* (EDI_285530), *Naegleria fowleri* (NdTy_078960), *Acanthamoeba castellanii* (ACA1_025690), *Cryptosporidium parvum* (cgd8_4330), *Cryptosporidium hominis* (Chro.80497), *Caenorhabditis elegans* (Q22053), *Neurospora crassa* (Q9HE26), *Schizosaccharomyces pombe* (P35551), *Candida glabrata* (Q6FN88), *Saccharomyces cerevisiae* (P15646), *Aedes aegypti* (A0A903UL91), *Drosophila melanogaster* (Q9W1V3), *Drosophila erecta* (Q8I1F4), *Taeniopygia guttata* (A0A674GVD4), *Falco tinnunculus* (A0A8C4XL37), *Anas zonorhyncha* (A0A8B9VAZ3), *Anas platyrhynchos* (A0A8B9T8F1), *Gallus gallus* (A0A8V0X119), *Coturnix japonica (*A0A8C2SSG7), *Xenopus tropicalis* (F6Z9G1), *Xenopus leavis* (P22232), *Pseudonaja textilis* (A0A670YR35), *Naja naja* (A0A8C6X751), *Anolis carolinensis* (H9GNL4), *Gopherus agassizii* (A0A452GP63), *Danio rerio* (Q7ZTZ4), *Carassius auratus* (A0A6P6R1J1), *Oreochromis niloticus* (I3JMN4), *Salmo salar* (B5X300), *Myotis lucifugus* (G1Q781), *Cavia porcellus* (A0A286XF99), *Capra hircus* (A0A452FJT7), *Bos taurus* (F1MM59), *Sus scrofa* (I3L5T3), *Mus musculus* (P35550), *Rattus norvegicus* (P22509), *Lodoxonta africana* (G3U1N0), *Equus caballus* (F6TIM4), *Gorilla gorilla* (G3RZC3), *Pan troglodytes* (H2QGB6), *Homo sapiens* (P22087), *Panthera leo* (A0A8C8X2B2), *Vulpes vulpes* (A0A3Q7UEI2), *Felis catus* (M3VZK8), *Panthera tigris* (A0A8C9JLA7), and *Ailuropoda melanoleuca* (G1M1Y1). All acquired sequences were aligned with the MEGA11 software (Tamura et al. 2021) using the MUSCLE algorithm. Subsequently, manual editing was performed after visual inspection. The aligned sequences were used to construct a phylogenetic tree using the maximum likelihood method. To assess the robustness of the nodes, a bootstrap consensus tree with 100 replicates and 10 random addition heuristic searches was constructed for each node considering the Jones-Taylor-Thornton substitution method.

### Protein modeling and docking

The three-dimensional (3D) model of *Giardia* fibrillarin was predicted using AlphaFold (Varadi et al. 2022), and visual representations were created using UCSF Chimera software (Pettersen et al. 2004). The quality of the resulting 3D structures was subjected to rigorous evaluation using PROCHECK (Laskowski et al. 1993), which involved meticulous examination of Psi/Phi angles within a Ramachandran diagram. For comparative purposes, the 3D model of human fibrillarin was also acquired (Protein Data Bank ID 7SE7). Docking studies between SAM and the predicted structure of *Giardia* fibrillarin were performed using SwissDock (Grosdidier et al. 2011) with the default parameters. The SAM structure (ZINC4214738) was obtained from the ZINC20 database (Sterling and Irwin 2015).

## Results

### Sequence analysis

*Giardia* fibrillarin, composed of 327 amino acids, has an estimated molecular weight of 35.24 kDa and an isoelectric point of 9.88. It is notable for its high glycine (15.6%) and arginine (11%) content. Predictive analysis suggests an instability index of 25.69, indicating a stable protein with a predicted half-life of more than 10 h.

**Fig. 1:**
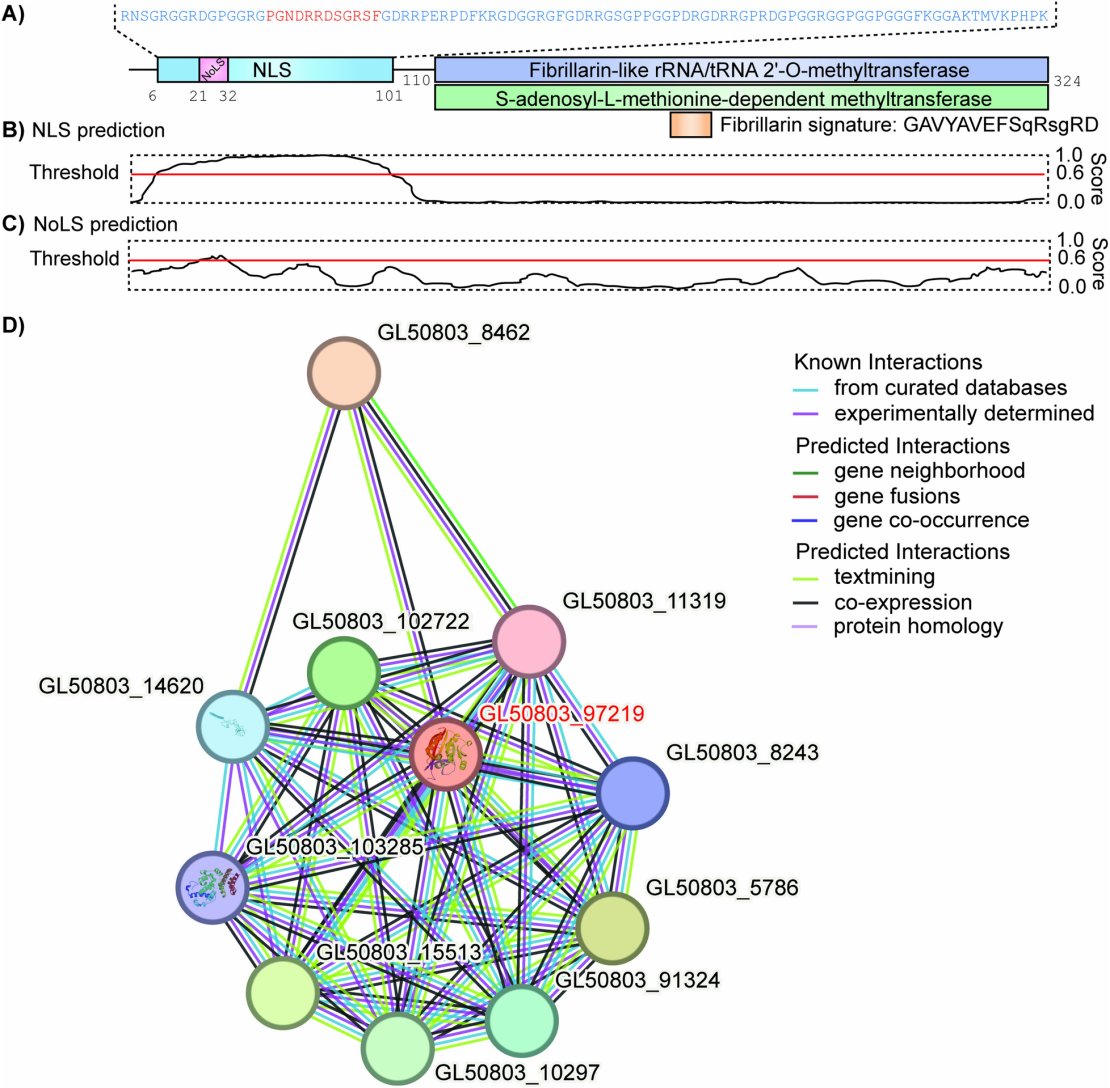
Analysis of *Giardia* fibrillarin sequence. (A) Domain analysis identified a glycine- and arginine-rich domain next to the SAM-dependent methyltransferase domain. (B) NLS prediction using NLStradamus (Nguyen Ba et al. 2009) pinpointed its location between amino acids 6 and 101. (C) NoLS prediction with NoD (Scott et al. 2011) identified its position between amino acids 21 and 32. (D) Protein–protein interaction networks functional enrichment analysis performed with the STRING database (Szklarczyk et al. 2021)

The *Giardia* fibrillarin sequence shows 51.81% identity and 62.95% similarity compared to human fibrillarin. When *Giardia* fibrillarin was contrasted with yeast fibrillarin, 51.31% identity and 63.85% similarity was observed. As might be expected, *Giardia* fibrillin orthologue is most closely related to that of *S. salmonicida*, another member of the order Diplomonadida, with 56.63% identity and 67.77% similarity. The SAM-dependent methyltransferase domain, which spans residues 110 to 324, has been identified in *Giardia* fibrillarin. It has a Rossmann folded structure, characteristic of this class of proteins (Fig. 1A). This region is the most conserved region of the protein, showing 59.91% identity and 74.77% similarity when compared to the human protein, and 57.08% identity with 72.96% similarity when compared to yeast. At the N-terminal end, a glycine- and arginine-rich domain, characteristic of eukaryotic fibrillarins, was identified (Fig. 1A). Notably, this domain exhibited an unusual abundance of aspartic acid (11%) and proline (13%) residues. This region is the most divergent between species. Since fibrillarin is a nucleolar protein with a NLS and a NoLS, these sequences were predicted in *Giardia* fibrillarin (Fig. 1A–C). The NLS of *Giardia* fibrillarin was predicted within the glycine- and arginine-rich domain, between amino acids 6 and 101. Furthermore, NoLS was identified between residues 21 and 32. Importantly, due to the divergence observed in *Giardia*, a lower threshold score was used for NoLS prediction, 0.6 instead of the default value of 0.8.

Since fibrillarin undergoes some post-translational modifications, an analysis was performed to predict the specific residues where these modifications might occur in *Giardia* fibrillarin. Multiple phosphorylation sites were predicted using three servers (Table 1). Based on consensus sites, *Giardia* fibrillarin is predicted to undergo phosphorylation at several sites: tyrosine 5 by TK (score 0.9810); serine 28 by CK1 (score 0.5074), PKA (score 0.738), PKC (score 0.725), or RSK (score 0.629); serine 57 by CAMK (score 0.0502) or PKA (score 0.790); and serines 202 (score 0.1277) and 205 (score 0.2257) by CK1. In addition, two acetylation sites were predicted: lysine 43, target of KAT2B (score 1.404), and lysine 325, target of CREBBP (score 1.371). Multiple methylation sites were also predicted for *Giaridia* fibrillarin at arginine residues (Table 2), with the highest scoring positions being 78 (score 0.999959), 55 (score 0.999507), 10 (score 0.999407), 19 (score 0.999223), and 69 (score 0.999127).

**Table 1:** Predicted phosphorylation sites in *Giardia* fibrillarin. S: serine, T: threonine, Y: tyrosine

**GPS 6.0**			
**Position**	**Amino acid**	**Kinase**	**Score**
57	S	CAMK	0.0502
28	S	CK1	0.5074
202	S	CK1	0.1277
205	S	CK1	0.2257
5	Y	TK	0.981
102	Y	TK	0.9601
127	Y	TK	0.9647
174	Y	TK	0.9585
197	Y	TK	0.9001
220	Y	TK	0.9252
**NetPhos 3.1**			
**Position**	**Amino acid**	**Kinase**	**Score**
28	S	–-	0.807
57	S	–-	0.757
31	S	–-	0.677
133	S	–-	0.574
119	S	–-	0.269
277	S	–-	0.224
8	S	–-	0.224
187	S	–-	0.168
151	S	–-	0.162
244	S	–-	0.144
139	S	–-	0.139
202	S	–-	0.124
276	S	–-	0.105
205	S	–-	0.096
280	S	–-	0.088
184	S	–-	0.087
272	S	–-	0.081
179	S	–-	0.073
108	S	–-	0.069
137	T	–-	0.467
281	T	–-	0.449
94	T	–-	0.43
117	T	–-	0.318
3	T	–-	0.309
217	T	–-	0.255
294	T	–-	0.249
181	T	–-	0.185
182	T	–-	0.173
193	T	–-	0.143
5	Y	–-	0.623
220	Y	–-	0.531
143	Y	–-	0.475
174	Y	–-	0.436
102	Y	–-	0.398
127	Y	–-	0.398
261	Y	–-	0.285
197	Y	–-	0.283
230	Y	–-	0.246
232	Y	–-	0.199
262	Y	–-	0.19
**PhosphoSVM**			
**Position**	**Amino acid**	**Kinase**	**Score**
8	S	–-	0.996
28	S	PKA PKC RSK	0.738 0.725 0.629
31	S	–-	0.996
57	S	PKA	0.790
133	S	–-	0.998
139	S	CKII	0.648
187	S	–-	0.876
202	S	–-	0.913
205	S	–-	0.995
272	S	PKC	0.779
276	S	–-	0.991
277	S	PKC	0.677
280	S	–-	0.884
3	T	PKC	0.703
94	T	PKC	0.680
281	T	–-	0.884
294	T	–-	0.978
5	Y	–-	0.903
143	Y	–-	0.723

**Table 2:** Predicted methylation sites in *Giardia* fibrillarin. R: arginine

Position	Amino acid	Score
78	R	0.999959
55	R	0.999507
10	R	0.999407
19	R	0.999223
69	R	0.999127
13	R	0.999058
49	R	0.998828
65	R	0.998089
72	R	0.997338
54	R	0.994732
26	R	0.982944
68	R	0.982285
44	R	0.980353
6	R	0.965493
36	R	0.964146
25	R	0.962064
111	R	0.952416
30	R	0.945959
207	R	0.823389
39	R	0.820079
35	R	0.795317
323	R	0.760142
131	R	0.729713
321	R	0.722644
260	R	0.662594
158	R	0.560912
204	R	0.508303

### Protein–protein interaction networks functional enrichment analysis

In an effort to analyze proteins that might be functionally related to *Giardia* fibrillarin, a protein–protein interaction networks functional enrichment analysis was performed. The generated map revealed 10 proteins closely associated with *Giardia* fibrillarin (Fig. 1D), including GL50803_11319 (U3 small nucleolar ribonucleoprotein IMP3), GL50803_8243 (Utp14 protein), GL50803_5786 (ribosome biogenesis protein NEP1), GL50803_91324 (U3 small nucleolar RNA-associated protein 20 containing C-terminal domain), GL50803_10297 (small nucleolar RNA-interacting protein U3), GL50803_15513 (WD repeat protein BING4), GL50803_103285 (HEAT repeat protein 1), GL50803_14620 (40S ribosomal protein S6), GL50803_102722 (ribosomal biogenesis protein BMS1), and GL50803_8462 (large ribosomal subunit protein eL27). Key processes identified in this network include rRNA methyltransferase activity, 90S pre-ribosomal processing, maturation of the small subunit (SSU)-rRNA from the tricistronic rRNA transcript (SSU-rRNA, 5.8S-rRNA, long subunit (LSU)-rRNA), U3 small nucleolar RNA (snoRNA) binding, the SSU processome, snoRNA binding, and the major rRNA processing pathway in the nucleolus and cytosol (Supplementary file 1).

### Phylogenetic analysis

A phylogenetic tree was constructed using the alignment of the fibrillarin orthologs of several species. Analysis of the constructed tree revealed that the *Giardia* fibrillarin, along with that of *S. salmonicida*, is closest to the orthologs of Archaea and parasitic amoebae (Supplementary Fig. 1). Thus, the analysis suggests that *Giardia* fibrillarin ranks as one of the most evolutionarily distant members within the eukaryote kingdom, indicating its unique evolutionary trajectory. It is noteworthy that the fibrillarin orthologs of parasitic amoebae appear to differ from those of free-living amoebae.

### Protein modeling

Since the complete three-dimensional (3D) structure of *Giardia* fibrillarin has not been determined by experimental methods, it was predicted using bioinformatics methods. Subsequently, an analysis of the predicted structure using Ramachandran diagrams demonstrated its suitability (Supplementary Fig. 2). Overall, the structure of *Giardia* fibrillarin was predicted to closely resemble that of the human protein, except for marked differences at its N-terminal end. This suggests that *Giardia* fibrillarin probably shares similar properties and functions to its human counterpart. The central domain of *Giardia* fibrillarin maintains a characteristic Rossmann fold, a structural motif commonly found in the central catalytic domains of most methyltransferases. This fold consists of a seven-stranded β-sheet (β1–β7) flanked by three α-helices on each side, with a total of six α-helices. All strands of the β-sheet, with the exception of the seventh strand, exhibit a parallel orientation. The antiparallel orientation of the seventh strand, located between the fifth and sixth, is considered crucial for the functionality of the domain. Analogous to human fibrillarin, it is proposed that the β1–β3 chains form the interaction site with SAM, whereas the β4–β7 chains participate in substrate binding. This proposal was validated by a docking analysis between SAM and the predicted structure of *Giardia* fibrillarin.

## Discussion

*Giardia* fibrillarin was initially identified in 1998 by Narcisi and collaborators (Narcisi et al. 1998b). However, few studies have been performed since then, resulting in a lack of comprehensive knowledge about the specific functions of this protein in *Giardia*. Although it is predicted to share similar functions with fibrillarins from other eukaryotes, primarily focused on pre-rRNA methylation, processing, and pre-ribosome assembly (Shubina et al. 2016), there is a possibility that this protein plays additional roles in the *Giardia* biology. For example, it could interact with small nucleolar RNAs (snoRNAs) and regulate, in some extent, their processing by DICER to generate miRNAs that contribute to antigenic variation (Lagunas-Rangel 2024a). Furthermore, given the proximity of telomeres to ribosomal DNA in *Giardia*, fibrillarin could interact with telomeres and telomerase proteins (Lagunas-Rangel 2024b). Furthermore, this protein could be considered a therapeutic target worthy of exploration, especially in light of the growing number of existing drug-resistant strains (Riches et al. 2020), which necessitates the discovery of new therapeutic strategies.

The protein–protein interaction networks constructed for *Giardia* fibrillarin mainly highlighted proteins associated with ribosome biogenesis, rRNA splicing, and rRNA methyltransferase activity. Undoubtedly, it is very likely that *Giardia* fibrillarin interacts with other nucleolar proteins. In this regard, a previous bioinformatics analysis revealed that the *Giardia* genome contains 216 nucleolar proteins orthologous to those found in higher eukaryotes such as *Saccharomyces cerevisiae*, *Arabidopsis thaliana*, and *Homo sapiens*. In addition, there are 39 *Giardia*-specific nucleolar proteins (Feng et al. 2020). Further study of these interactions could provide valuable information on the intricate network of *Giardia* nucleolar functions. Experimentally, it has been established that *Giardia* fibrillarin can bind and co-localize with small nuclear RNAs (snRNAs) to form a ribonucleoprotein particle (RNPP) complex, particularly with snRNAs, RNA J, RNA H, and RNA D (Ghosh et al. 2001; Ganguly et al. 2004).

Given the apparent absence of assembly and disassembly of the nucleolus in *Giardia* (Lara-Martínez et al. 2016), coupled with its semi-open mitosis (Sagolla et al. 2006; Lagunas-Rangel et al. 2021), an intriguing avenue of exploration is to understand the dynamic behavior of fibrillarin throughout the cell cycle. Furthermore, investigating its role during encystment and excystment, which are associated with the activation and deactivation of several metabolic processes (Kim et al. 2009; Birkeland et al. 2010; Jiráková et al. 2012), would provide valuable information on the regulatory mechanisms governing these crucial transitions. During these dynamic processes, fibrillarin activity may be intricately regulated, and post-translational modifications, such as phosphorylation, methylation, and acetylation, are likely to play key roles (Iyer-Bierhoff et al. 2018). The residues predicted here for these modifications provide insights into possible regulatory mechanisms. Experimental validation is crucial to understand how these modifications influence the role of *Giardia* fibrillarin in dynamic cellular processes.

Fibrillarin, characterized by its high degree of conservation, serves as a valuable tool for exploring phylogenetic relationships between organisms with considerable evolutionary distance (Rodriguez‐Corona et al. 2015; Shubina et al. 2016). To unravel these connections, a phylogenetic tree was meticulously constructed using the maximum likelihood method. Maximum likelihood methods outperform parsimony methods by allowing the estimation of the evolutionary history pattern while incorporating the probabilities of character state changes from a specific evolutionary model (Nixon 2001). Phylogenetic analysis revealed that *Giardia* fibrillarin exhibits a closer evolutionary affinity to fibrillarin orthologs found in archaea and parasitic amoebae. This places *Giardia* fibrillarin as one of the most distant members of the eukaryotic kingdom. It is worth mentioning that *Giardia*, once considered a “primitive” or “early branching lineage” organism, has been reconsidered in recent studies (Islas-Morales et al. 2021). These investigations propose that the evolutionary position of *Giardia* is more accurately attributed to reductive evolution than to early branching (Lloyd and Harris 2002; Burki et al. 2020).

The predicted structure of *Giardia* fibrillarin closely resembles that of its human counterpart, although with notable differences at the N-terminal end. SAM occupies a position similar to that of the human protein, establishing interactions with the β1–β3 chains (Shubina et al. 2016). This suggests that the development of competing SAM inhibitors specific for *Giardia* fibrillarin could prove valuable for further investigation. These molecules would not only contribute to deciphering the intricacies of the unique biology of *Giardia* fibrillarin, but could also shed light on potential regulatory checkpoints and molecular events that could be targeted for therapeutic intervention.

## Supplementary Information

Below is the link to the electronic supplementary material.

Supplementary file1 Phylogenetic tree of fibrillarin orthologs. Using the aligned sequences of fibrillarin orthologs in various species, phylogenetic distances were calculated using the maximum likelihood algorithm. The percentage of replicate trees in which the associated taxa clustered in the bootstrap test is indicated next to the branches (TIF 23631 KB)

Supplementary file2 Structural analysis of Giardia fibrillarin. A) Predicted structure of Giardia fibrillarin by AlphaFold. B) Structure of human fibrillarin. C) Overlay of images representing predicted Giardia fibrillarin (gray) and human fibrillarin (blue) for comparative analysis. D) Docking analysis between SAM and the predicted Giardia fibrillarin structure. E) Ramachandran plot of the predicted structure of Giardia fibrillarin (TIF 69695 KB)

Supplementary file3 (XLSX 14 KB)

## Data Availability

The data that support the findings of this study are available from the corresponding author upon reasonable request.
