# Supplementary figures and images for: Giardia fibrillarin: a bioinformatics exploration of sequence and structure
Source: J Appl Genet. 2024 Nov 11;66(1):241–8. doi: 10.1007/s13353-024-00920-w (PMC11761994; doi:10.1007/s13353-024-00920-w)

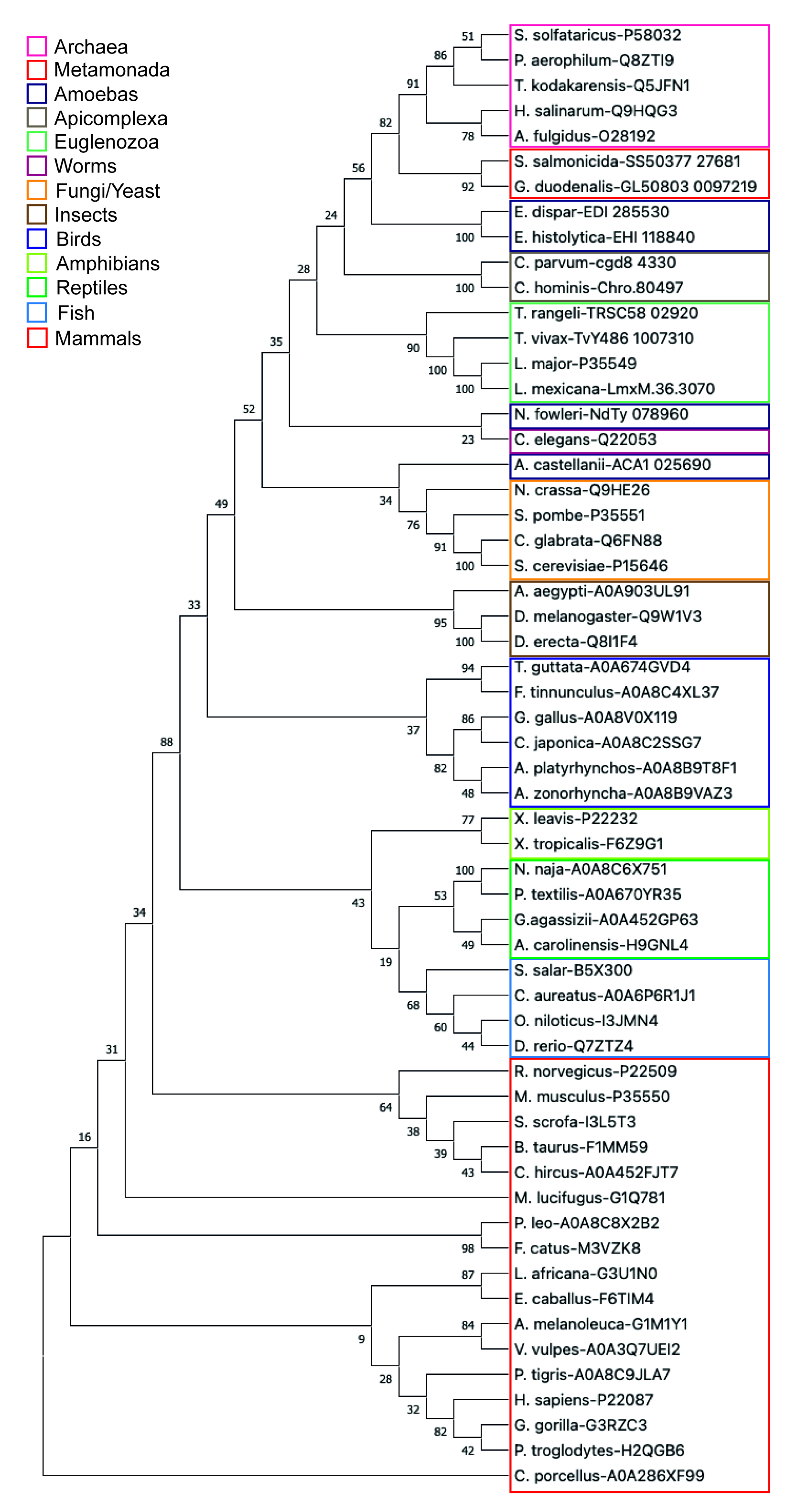

Supplement: Supplementary file 1 — Supplementary file1 Phylogenetic tree of fibrillarin orthologs. Using the aligned sequences of fibrillarin orthologs in various species, phylogenetic distances were calculated using the maximum likelihood algorithm. The percentage of replicate trees in which the associated taxa clustered in the bootstrap test is indicated next to the branches (TIF 23631 KB) [file 13353_2024_920_MOESM1_ESM.tif]
